# A 10-year comparison of short versus long-term court-ordered psychiatric hospitalization: a follow-up study

**DOI:** 10.1186/s13584-023-00561-0

**Published:** 2023-04-20

**Authors:** Daniel Argo, Khaled Daibas, Igor Barash, Moshe Z. Abramowitz

**Affiliations:** 1 Jerusalem Mental Health Center, Eitanim Psychiatric Hospital, Jerusalem, Israel; 2 Mazor Mental Health Center, Acre, Israel; 3 Peres Academic Center, Rehovot, Israel; 4 Hebrew University-Hadassah School of Medicine, Jerusalem, Israel

**Keywords:** Involuntary hospitalization, Outcome, Mental health management

## Abstract

**Background:**

The Israel Mental Health Act of 1991 stipulates a process for court-ordered involuntary psychiatric hospitalization. As in many Western countries, this process is initiated when an individual is deemed “not criminally responsible by reason of mental disorder (NCR-MD)” or “incompetent to stand trial (IST).” A patient thus hospitalized may be discharged by the district psychiatric committee (DPC). The decision rendered by the DPC is guided by an amendment to the Mental Health Act that states that the length of the hospitalization should be in accordance with the maximum time of incarceration associated with the alleged crime. Little empirical research has been devoted to the psychiatric, medical, and social outcome of short versus long-term hospitalization under court order.

**Methods:**

In our study we examined the outcomes of court-ordered criminal commitments over a 10-year period (2005–2015) at the Jerusalem Mental Health Center with a catchment area of 1.5 million. We found 136 cases (between the ages of 18 and 60) of criminal commitments during that period and used the average length of hospitalization, 205 days, as a cutoff point between short and long stays. We compared the outcomes of short and long hospitalizations of discharged patients using a follow-up phone survey (at least 7 years post-discharge) and data extracted from the Israel National Register to include recidivism, patient satisfaction and trust in the system, readmission, and demise.

**Results:**

We found no statistically significant difference between short-term and long-term hospitalizations for reducing instances of re-hospitalization (*p* = 0.889) and recidivism (*p* = 0.54), although there was a slight trend toward short-term hospitalization vis-à-vis reduced recidivism. We did not find a statistical difference in mortality or incidents of suicide between the two groups, but the absolute numbers are higher than expected in both of them. Moreover, our survey showed that short-term hospitalization inspired more trust in the legal process (conduct of the DPC), in pharmacological treatment satisfaction, and in understanding the NCR-MD as a step toward avoiding future hospitalization and that it resulted in a higher level of patient satisfaction.

**Conclusions:**

The results we present show that as far as recidivism and readmission are concerned, there is no evidence to suggest that there is an advantage to long-term hospitalization. Although there may be unmeasured variables not investigated in the present study that might have contributed to the discrepancy between long- and short-term hospitalization, we believe that longer hospitalizations may not serve the intended treatment purpose. Additionally, the high cost of long-term hospitalization and overcrowded wards are obviously major practical drawbacks. The impact of the clinical outcomes should be reflected in medico-legal legislation and in court-ordered hospitalization in particular.

## Background

What is the proper time frame for an individual deemed not criminally responsible by reason of mental disorder (NCR-MD) or incompetent to stand trial (IST) to remain in a forensic psychiatric inpatient setting? In the Israeli system, virtually all individuals ordered into such a forensic setting are deemed NCR-MD and in some cases they are also rendered IST, in addition to being NCR-MD. It is rare to find in the Israeli system a person regarded as only IST. The reason for this is that an individual identified as IST is automatically sent for hospitalization, and under Israeli law can theoretically be prosecuted when he/she is deemed competent to stand trial. However, to date there have been no instances of an IST designate eventually standing trial, so such individuals are ultimately considered NCR-MD.

In 2019, a total of 545 individuals were hospitalized in Israel by court order under the Israeli Mental Health Act of 1991 [1], of which 512 were readmissions [2]. This law explicitly states that the goal of incarceration including court-ordered cases is treatment. Indeed, the literal translation of the title of “the Israel Mental Health Act” is “the Law for the Treatment of the Mentally Ill.” However, recent amendments stipulate that the length of the hospitalization should not exceed the maximum time of incarceration associated with the alleged crime.

The district psychiatric committee (DPC), made up of two psychiatrists and headed by an attorney at the level of a magistrate, is authorized to decide on discharge or conditional release from the hospital; an amendment to the law passed in 2014 [1] serves as a guideline to prevent early release of a potentially dangerous individual. This amendment created a link between the type of offense committed and the length of hospitalization, which had previously been determined solely on the basis of the individual’s psychiatric condition. Thus, a person who commits a minor crime while psychotic (e.g., steals food) and quickly recovers during in-hospital treatment can remain incarcerated for years according to the maximum sentence for the offense, despite his/her clinical psychiatric remission.

Much has been written about both the ethical issues of restricting patients’ rights and limiting their freedom [3–5] and the arguments around the decision to discharge from hospitalization [6, 7]. However, less has been written regarding the outcome of court-ordered criminal commitments. Is long-term hospitalization more efficacious than short-term? The answer to this question has considerable medical, ethical, and economic implications.

## Method

This outcome study was based on data from the Israeli Ministry of Health’s National Psychiatric Hospitalization Registry, which contains complete information on all psychiatric admissions in Israel since 1950 [8]. Approximately 22,000 hospitalizations are recorded annually. We evaluated the outcomes for discharged court-ordered hospitalizations from the Jerusalem Mental Health Center over a 10-year period (2005–2015) with a minimum follow-up period of 7 years. The Jerusalem Mental Health Center is the principal inpatient mental health facility in the region with two campuses, a catchment area of 1.5 million, and a diverse, multilingual Jewish and Arab population. Individuals older than 60 and younger than 18 were not included in the study. Some of the patients were discharged and hospitalized again during the 10-year period. We found 136 cases of court-ordered criminal commitment, NCR-MD (with or without an additional determination of IST), that met the criteria during that period and used the average length of hospitalization, 205 days, as a cutoff point between short- and long-term hospitalization (the average duration of 203.57 days was rounded off to 205; the median duration was 207 days).

**Fig. 1:**
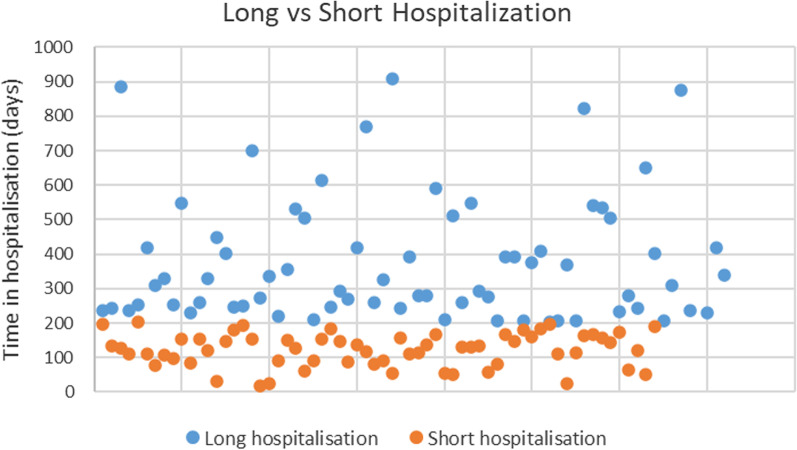
A single extreme value of 1679 was not included in the figure to simplify the presentation

We then extracted information on the two groups: short-term and long-term (N = 64, average length of hospitalization 122 days and N = 72, average length of hospitalization 402 days). A scatterplot visualization of the distribution can be seen in Fig. 1. As demonstrated, the subjects’ distribution is significant and not clustered.

Telephone interviews in Hebrew, Arabic, or English were conducted with the discharged individuals or first-degree relatives between January and April 2022. The study obtained Institutional Review Board approval from the Jerusalem Mental Health Center and informed consent from all the participants. The interviews consisted of an inpatient satisfaction questionnaire using adapted relevant themes in accordance with the work of Macinnes, Beer, et al. [9] and similar to a Ministry of Health questionnaire that was last posted in 2015 [10], as well as questions relating to events post-discharge (e.g., recidivism). The response rate was 73% and 77% for the short-term and long-term hospitalizations, respectively (see Table 1). In four cases of short-term hospitalization and six of long-term hospitalization, the data about the patient were received from a family member.

**Table 1:** Recruitment of discharged court-ordered criminally committed patients (from 2005–2015) to the follow-up (2022)

	Short hospitalization (less than 205 days)	Long hospitalization (more than 205 days)
Number of patients	64	72
Response rate*	44 (73%)	51 (77%)
Refused to participate or could not be reached	17 (27%)	15 (21%)
Deceased (non-suicide)	2 (3%)	3 (4%)
Committed suicide	1 (1.5%)	1 (1.5%)
Arrested/ in custody	1 (1.5%)	1 (1.5%)
In closed rehabilitation facility		1 (1.5%)

*Response rate calculated only of those patients who are alive and not arrested or institutionalized

Readmissions were extracted from the Israel National Registry and compared between the two groups.

### Statistical analysis

Statistical significance was set at *p* < 0.05 level. We used a chi square test for simple variables and Fisher’s Exact Test to assess the connection between the length of hospitalization and re-hospitalization. Demographics (age, gender, and marital status), diagnosis, length of hospitalization, and type of offense are presented in Table 2.

**Table 2:** Demographic data, diagnosis, and type of offense comparison between short- and long-term court-ordered hospitalization

Short Hospitalization (N = 64)	Long Hospitalization (N = 72)	*p* value
*Age*		
34	37	0.1
*Marital status*		
Single—56%	Single—63%	0.49
Married—22%	Married—22%	
Divorced—22%	Divorced—14%	
	Widower- 1%	
*Gender*		
Male—91%	Male- 94%	0.39
Female—9%	Female—6%	
*Nationality*		
Jewish—71%	Jewish—76%	0.55
Arab—28%	Arab—24%	
*Diagnosis*		
Schizophrenia—75%	Schizophrenia—86%	0.18
Schizophrenia and drug abuse—8%	Schizophrenia and drug abuse—10%	
Bi-polar—9%	Bi-polar—1%	
Personality dis.—5%	Personality dis.—1%	
Delusional dis.—1.5%		
*Criminal offense*		
Minor Violence—68%	Minor Violence—56%	0.39
Threats—16%	Threats—18%	
Inappropriate Behavior in public place—8%	Inappropriate Behavior in public place—10%	
Theft—3%	Theft—1%	
Damage to property– 1.5%	Damage to property—3%	
Sexual offences—1.5%	Sexual offences—5.5%	
Drugs—1.5%	Murder—1%	
*Average length of hospitalization (days)*		
122	402	

In statistical terms, the two groups studied were essentially the same population.

## Results

Statistically significant differences were found between the short- and long-term hospitalization groups in the trust in/conduct of the DPC, in patient satisfaction, satisfaction with the pharmacological treatment, and in the answer to whether the hospitalization helped to prevent the individual from relapsing. Other issues such as trust in the legal aid provided by the Ministry of Justice came close to statistical significance. There was a trend toward significance in the survey questions dealing with the satisfaction regarding psychological and psychiatric treatment, but our sample was too small to reach a conclusion.

Table 3 sums up the results of the follow-up and survey.

**Table 3:** Minimum 7-year follow-up comparison between short- and long-term court ordered hospitalization

	Short hospitalization (205 less than days)				Long hospitalization (205 more than days)				*p* value
	Very/Satisfied	Neutral	Very/Not satisfied	Don’t Know	Very/Satisfied	Neutral	Very/Not satisfied	Don’t know	
Psychiatric treatment	81%	11%	7%		68%	17%	13%		0.35
Pharmacological treatment	90%	5%	5%		68%	10%	21%		**0.028**
Legal representation	74%	9%	16%		53%	31%	41%	2%	0.056
Trust in/conduct of the DPC	70%	11%	18%		27%	31%	41%		**0.0002**
Treatment by nurses	81%	11%	7%		75%	14%	11%		0.67
Psychological treatment	83%	11%	5%		68%	22%	8%	2%	0.29
General satisfaction	81%	5%	14%		57%	17%	25%		**0.03**
	Too long	appropriate	Too short	Don’t know	Too long	appropriate	Too short	Don’t know	
Length of hospitalization	46%	51%	0	2%	86%	14%			**0.00006**
	Greatly	Helped a bit	Not at all		Greatly	Helped a bit	Not at all		
Did the hospitalization helped to avoid similar situations?	86%	11%	2%		35%	49%	15%		** < 0.00001**
	Yes	No	Refuse to answer		Yes	No	Refuse to answer		
Arrests after the discharge	23%	74%	2%		30%	70%			0.54

The statistical significance of *P* < 0.05 is highlighted in bold

**Fig. 2:**
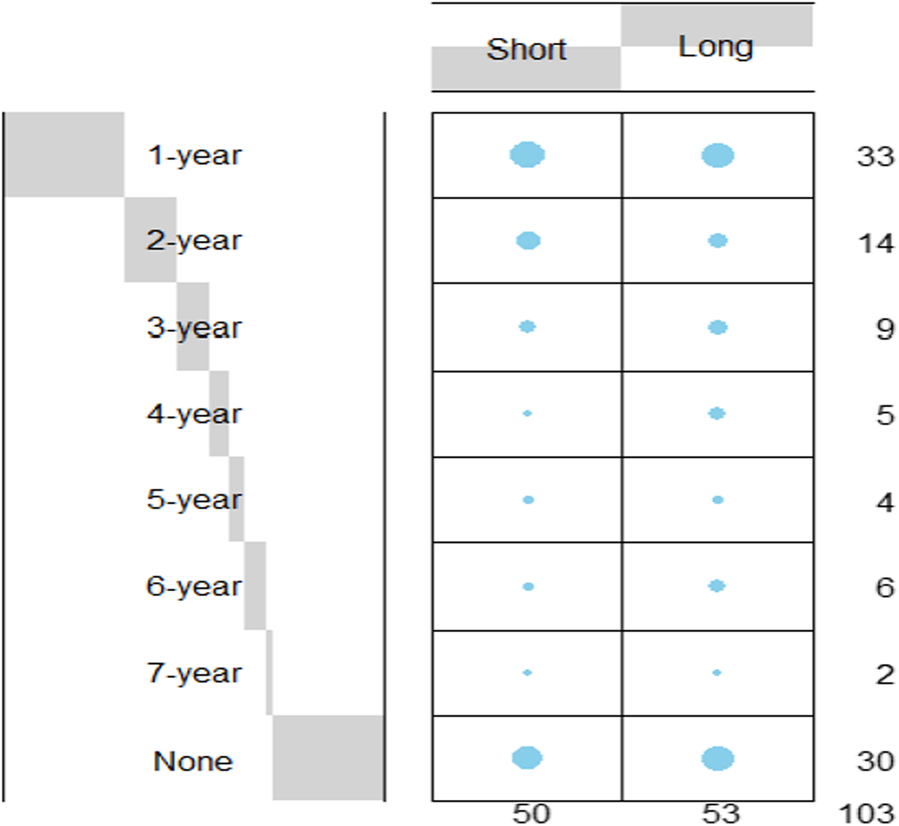
Balloon plot visualizing shared distribution between the length of index hospitalization and re-hospitalization between the short- and long-term groups over a 7-year follow-up period

As our study population included patients discharged between 2005 and 2015, there was a minimum of 7 years from discharge to follow-up. Instances of re-hospitalization were extracted from the National Registry. A balloon plot visualizing multivariate categorical data demonstrates shared distribution between the duration of the index (first) hospitalization stay and survival between the short- and long-term groups over a 7-year follow-up period (Fig. 2).

We did not find a statistical correlation between the duration of the index hospitalization and the rate of re-hospitalizations in either group (*p* = 0.889).

## Discussion

Aside from the moral and legal issues pertaining to the incarceration of NCR-MD individuals, there remains the question as to whether patients actually benefit from being hospitalized. Since the enactment of the 19th-century Mc’Naughton rules, it has been widely accepted that mental disability should be a necessary condition for negating criminal responsibility [11, 12]. In the United Kingdom, the court must issue a hospital order in cases punishable by imprisonment according to Sect. 37 of the Mental Health Act of 1983 [13]. If the person no longer experiences symptoms of mental disorder, he/she can be discharged by a tribunal, even if there is a strong possibility that he/she might relapse and offend again. When there is sufficient evidence that the patient poses a particularly high risk to other people if released, has a pronounced history of dangerous behavior, or has committed a particularly serious offence, Sect. 41 is used in conjunction with a restriction order limiting discharge and other rights [13].

In Canada, there must be clear evidence of a significant risk to the public before a court or a review board can maintain control over an accused through the imposition of a conditional discharge or detention order [14].

In Israel, such guidelines and restrictions were only loosely specified until an amendment to the Mental Health Act that limited a court-ordered psychiatric hospitalization to the maximum sentence for the crime committed was passed in 2014 [1]. Though not intended by the legislature (the amendment’s preamble specifically states that the intention is only to set an upper limit to NCR-MD hospitalization, not to mandate that the DPC maintain criminal commitment for the length of that limit), DPCs continue to be reluctant to release such patients prematurely. This may be due to the possible negative public perception of having individuals who commit murder while psychotic, for example, “getting off” with only a short court-ordered criminal commitment and being discharged soon after remission.

Our study set out to examine the efficacy of short- and long-term NCR-MD hospitalizations with a minimum 7-year follow-up. We found no significant statistical difference in terms of age, gender, marital status, nationality, psychiatric diagnosis, and offense committed between the short- and long-term hospitalization groups whether the cutoff was the average or median length of hospitalization.

There was no statistically significant difference between short- and long-term hospitalization in reducing occurrences of re-hospitalization (*p* = 0.889) and recidivism (*p* = 0.540). Consequently, there is apparently no advantage to long-term hospitalization in that regard. Moreover, in our follow-up survey, the short-term group showed a clear advantage over the long-term group in patient satisfaction and in understanding the NCR-MD as a step toward avoiding future hospitalization. There was also satisfaction with the pharmacological treatment in this group, a crucial factor for determining the prognosis and the success of rehabilitation programs. We also found more trust in the conduct of the DPC in the short-term group, a positive development considering the distrust frequently encountered in the NCR-MD population.

The two groups were not statistically different in terms of mortality and suicide, but it should be noted that the absolute numbers were higher than expected in both groups. It may be that our patient population over the 10-year span was too small a sample to detect any such differences between them, but it would seem that a review of the long-term hospitalizations in our study provides little evidence for the rationales of preventing relapse and re-offending. There was also no evident statistical advantage in regard to the remaining major justification for a long-term inpatient stay, the treatment of mental illness—the underlying principle of the current law.

The high cost of long-term hospitalization is obviously a major practical drawback. Aside from the per diem cost of an inpatient stay, it is difficult to overestimate the effect of admitting court-ordered patients to the already overcrowded acute wards in Israel [15]. Apart from other difficulties, such overcrowding leads to increased incidences of violence on the part of patients toward psychiatric health care workers [16]. Moreover, our study did not confirm any cost-effective benefit (i.e., reduced re-hospitalization) in long-term hospitalization despite the major monetary investment.

The outcomes for court-ordered criminally committed hospitalizations should be studied by the psychiatric and legal communities so that fully informed decisions can be made as to what is most beneficial for the mentally ill patient as well as for society and to determine the best time to discharge an individual from inpatient care and to initiate community-based court oversight [17].

By way of a disclaimer, it should be noted that the present paper does not propose to compare outcomes before and after the latest amendment (the period studied, 2005–2015, precedes its implementation). Rather, it hopefully may serve to provide pertinent data as to whether there are (treatment) advantages to shortening psychiatric hospitalization of NCR-MD individuals. Moreover, the option of continued hospitalization to complete the psychiatric treatment can be achieved at the discretion of the district psychiatrist who has the power (using a civilian commitment order) to admit a mentally ill patient to a psychiatric ward against his/her will.

It is important to state that although we found no statistically significant differences between the groups in the basic demographic data, diagnosis, and type of offense charged, there may be other “hidden” factors that differ, which were not investigated in the present study (e.g., family and social support, response to treatment, personality traits of the individuals, the exact composition of the DPC among others). This study was designed to demonstrate the discrepancy in the length of hospital stay, but clearly additional research is needed to clarify precisely which determining factors are chiefly in play.

### Policy implications

On a broader note, the authors advocate the use of follow-up and outcome studies in reviewing legislative changes to mental health laws [6]. The present study will hopefully provide such evidence-based data regarding the efficacy of short- and long-term criminal commitments. Many existing reviews on the subject serve well to tell us more about the smooth running of the system and the application of the law in view of patients’ rights [18, 19] but leave us not knowing whether new legislation enhances patients’ benefits from treatment. Outcome studies using similar methodology as one would use comparing a novel to an existing medical procedure are much needed in the field of mental health management.

The impact of clinical outcomes should be reflected in medico-legal legislation and in court-ordered hospitalization in particular. Long-term implications such as post-discharge morbidity and mortality, patient satisfaction, and continued adherence to treatment should be taken into account at all levels of decision-making, not only those by treating psychiatrists.

## Conclusions

The results we present fail to demonstrate any statistically significant difference between short- and long-term hospitalization in reducing occurrences of re-hospitalization (*p* = 0.889) and recidivism (*p* = 0.540), nor did we find any difference in terms of mortality and suicide in the follow-up.

Furthermore, the short-term group showed a clear advantage over the long-term group in patient satisfaction, in understanding the NCR-MD as a step toward avoiding future hospitalization, in satisfaction with the pharmacological treatment, and in patient trust in the conduct of the DPC. Further research is needed to provide data on parameters that were not scrutinized in the present study.

Unfortunately, despite repeated efforts, we were unable to obtain data from official sources concerning arrest records and the offenses charged. Response rates for discharged court-ordered criminally committed patients were calculated from those patients alive and not arrested or institutionalized. The authors decided on the next best thing—asking for a self-report from patients (or from first-degree relatives, typically knowledgeable whether the committed patient was arrested or institutionalized). Admittedly this is a limitation, although this new information provides an idea of what the true trend of recidivism might be.

The authors advocate adapting an approach of systematic inquiry over a period of years regarding the clinical and rehabilitation outcomes of medico-legal decisions to better improve the mental health system.

## Data Availability

The data that support the findings of this study are available from the Israeli Ministry of Health, but restrictions apply to the availability of these data, which were used under license for the current study and are not publicly available. Data are, however, available from the authors upon reasonable request and with permission of Israeli Ministry of Health.

## Abbreviations

IPH: Involuntary psychiatric hospitalization
NCR-MD: Not criminally responsible by reason of mental disorder
DPC: District psychiatric committee
IST: Incompetent to stand trial
